# Motivations to use hormonal contraceptive methods and condoms among HIV-positive and negative women randomized to a progestin contraceptive in Malawi: a qualitative study

**DOI:** 10.1186/s12905-021-01236-1

**Published:** 2021-03-20

**Authors:** Agatha K. Bula, Kendra Hatfield-Timajchy, John Chapola, L. Chinula, Stacey A. Hurst, Athena P. Kourtis, J. H. Tang

**Affiliations:** 1 UNC Project-Malawi, Private Bag A-104, Lilongwe, Malawi; 2 U.S. Centers for Disease Control and Prevention, Atlanta, GA USA; 3 Department of OB-GYN, University of North Carolina At Chapel Hill, Chapel Hill, NC USA

**Keywords:** Depot medroxyprogesterone acetate, Levonorgestrel implant, Motivation, Condom use, Contraceptive use, Malawi

## Abstract

**Background:**

Although many countries have been promoting hormonal contraceptives to prevent unintended pregnancy and condom use to prevent HIV transmission, little is known about how women targeted by these messages have interpreted and internalized them. We describe HIV-positive and negative women’s understanding of the benefits of contraception and condoms and their motivations to use them.

**Methods:**

This is a qualitative sub-study from a clinical trial evaluating the effects of progestin contraception on HIV-positive and negative women aged 18–45 years randomly assigned to depot medroxyprogesterone acetate (DMPA) injection or levonorgestrel (LNG) implant. We purposively recruited 41 women to participate in in-depth interviews (IDIs) and focus group discussions (FGDs) after randomization into the main study. We conducted a total of 30 IDIs and 6 FGDs comprised of 4–7 women (N = 32). All women were counselled about potential risks for HIV acquisition/transmission with progestin-only contraception, drug-drug interactions between the implant and efavirenz-based ART, and the need to use condoms with their assigned contraceptive to help prevent pregnancy and HIV acquisition and transmission.

**Results:**

All women understood that HIV is transmitted through unprotected sex and that HIV transmission can be prevented through condom use but not DMPA injection or LNG implant use. Nearly all HIV-positive women knew or suspected that their partners were also HIV-positive and were most interested in using condoms to prevent infection with a drug-resistant HIV strain to keep their HIV viral load low. Almost all reported that their partners agreed to condom use, but few used them consistently. Most women believed that condoms were effective at preventing both HIV and pregnancy if used consistently. Nearly all women considered contraception and condom use as important in preventing unintended pregnancy and HIV because partner disclosure of HIV status is low.

**Conclusion:**

Our results showed that both HIV-positive and negative women understood modes of HIV transmission and prevention and were aware that hormonal contraceptives are only effective for preventing pregnancy and not HIV. Although both HIV-negative and positive women were motivated to use condoms to prevent both HIV acquisition and infection with other HIV strains respectively, they all faced challenges from their partners in using condoms consistently.

**Supplementary Information:**

The online version contains supplementary material available at 10.1186/s12905-021-01236-1.

## Background

In 2017, an estimated 300,000 women died worldwide due to complications during pregnancy and childbirth. This translates into a global maternal mortality ratio (MMR) of 211 deaths per 100,000 live births, a 38% reduction between 2000 and 2017 [1–3]. However, sub-Saharan Africa (SSA) continues to have the highest annual MMR of 542 deaths per 100,000 live births, accounting for 65% of maternal deaths globally [2, 4]. HIV infection is classified as an indirect cause of death among women of reproductive age in SSA, where more than 90% of pregnant women living with HIV reside [5–8].

Contraception has profound health benefits, principally by reducing the risks of unintended pregnancy and by allowing for optimal spacing between pregnancies, which contributes to safer pregnancies and heathier infants [3]. An estimated 218 million women of reproductive age (15–49) worldwide, however, have unmet need for contraception, and the prevalence remains low in much of sub-Saharan Africa [9]. Among women using reversible contraceptives, discontinuation rates are high (38% by the 12th month and 64% by the 36th month), despite the fact that only 6–11% discontinue after 12 months because they want to get pregnant [10, 11]. Findings from a randomized, multi-centre, open-label trial across 12 research sites demonstrated that the three contraceptive methods (DMPA-IM, a copper intrauterine device (IUD), and a levonorgestrel (LNG) implant) under trial were highly effective, and the differences in HIV acquisition risk across methods did not exceed 50% [12]. Little is known about how women have understood these risks and if they have impacted contraceptive discontinuation.

In Malawi, the MMR is one of the highest in the world, estimated at 439 per 100,000 live births [13]. This is due in part to a high total fertility rate (TFR), estimated at 4.4 children per woman, and high HIV prevalence (13% among women aged 15–49 years) [13]. Although 33% of married Malawian women want to wait at least 2 years before having another child and 49% do not want any more children, only 58% of them currently use modern methods of contraception [13]. The decision to initiate and continue use of a contraceptive method involves multiple factors, primarily the acceptability and accessibility of method options, combined with fertility desire. High contraceptive discontinuation rates for reasons other than pregnancy desire, such as side effects, health concerns, partner disapproval, and misconceptions, are a public health concern because unintended pregnancy is associated with the previously-noted adverse maternal and infant outcomes [14]. Therefore, the primary objective of our paper is to describe women’s understanding of their HIV acquisition risk, views on the effectiveness of hormonal contraceptive methods and condoms, including among women using antiretroviral therapy (ART), and motivations to use contraceptive methods and condoms.

## Materials and methods

### Study type and design

This qualitative study collected data using both individual in-depth semi-structured interviews (IDIs) and focus groups discussions (FGDs). One-on-one sessions provided opportunities to collect sensitive information about individual experiences, perspectives about HIV acquisition, contraceptive uptake and condom use in a private setting and for the interviewer to tailor probes about motivators and barriers to contraceptive and condom use. We used FGDs to explore the women’s collective perspectives about HIV acquisition, contraceptive uptake and condom use.

### Study Setting and population

We recruited participants to participate in the sub-study from a randomized clinical trial evaluating the effects of progestin contraception on HIV shedding and immune activation in the genital tract among 97 women aged 18–45 years (73 HIV-positive and 24 negative) [15]. Women were randomly assigned to the DMPA or 5-year levonorgestrel implant (LNG) from May 2014 to April 2015 at Bwaila Hospital, a government hospital in Lilongwe, Malawi with over 15,000 deliveries annually. Trial participation lasted for up to 33 months after contraceptive initiation.

Prior to study enrollment from the family clinic, interested women received comprehensive family planning education from a study nurse using the Malawi Ministry of Health family planning counselling flipbook that had information about all available contraceptive methods in Malawi, including information on the DMPA injection and LNG implant. Women who were interested in enrolling in the study then underwent screening, an informed consent process, and enrollment into the trial [15]. During the consent process, all women received information about the potential risk of HIV acquisition and transmission with hormonal contraception and the importance of condom use for HIV prevention. Women who desired hormonal contraception and met the study eligibility criteria (no plans to become pregnant within the next year, confirmed HIV status and willing to be placed on either DMPA injection or LNG implant) were randomized to receive either the DMPA injection or LNG implant at a future visit and counseled about the potential side effects of their assigned contraceptive before and after initiating it. At each main study visit, study staff also counselled participants about the potential risk of HIV acquisition and transmission with hormonal contraception and the need to use condoms with their assigned contraceptive to prevent HIV acquisition and transmission.

In December 2014, we submitted an amendment to the IRBs to update our Study Protocol, consent forms, Implant Counselling Aid, and end-of-visit Counselling Script to include recently-published information about the potential decreased effectiveness of the levonorgestrel implant among HIV-positive women on ART and the need to use condoms for back-up contraception.^1^ Three separate Implant Counselling Aids were developed to ensure that women could make informed decisions about: 1) whether to enrol in the study (if they were undergoing study screening, known as the “Pre-Enrolment Implant Counselling Message”), 2) whether they wanted to continue in the study (if they had enrolled but not undergone randomization, known as the “Pre-Randomization Implant Counselling Message”), and 3) whether they wanted to continue with the implant (if they had already been randomized to the implant, known as the “Post-Randomization Implant Counselling Message”). Amendment approval from all IRBs was received by the end of March 2015, and the revised documents were implemented immediately thereafter. The “Pre-Enrolment Implant Counselling Message” was never used as all women had already been enrolled in the study by the time IRB approval was received. However, the “Pre-Randomization Implant Counselling Message” and “Post-Randomization Implant Counselling Message” were administered to appropriate women, depending on their HIV status and whether they had been randomized or not at the time of IRB approval. Women who were HIV-negative and women already randomized to DMPA did not receive any of these “Implant Counselling Messages” since the messages did not apply to them.

### Data collection

For this qualitative sub-study, we recruited women during the last year of the study follow-up period to participate in IDIs and FGDs using purposive sampling. Experienced qualitative researchers JC and AB conducted all IDIs and FGDs. The 30 IDIs took place in a private room at the clinic at Bwaila Hospital, and the 6 FGDs took place in a private conference room on the campus of Kamuzu Central Hospital in Lilongwe. Forty-one women participated in either an IDI or FGD. At enrolment in the main study, 28 of these women were randomized to the LNG implant and 13 were randomized to DMPA.

Women who took part in the IDIs and FGDs were categorized into three different categories described below based on the counselling messages they received during the main study, which method they were randomized to, and their HIV status. The 19 HIV-negative women included in our sub-study from Category A only received messages about the risk of HIV acquisition and transmission with hormonal contraception since the “Implant Counselling Messages” did not apply to them. Sixteen of the HIV-positive women included in our sub-study from Category B received the “Pre-randomization Implant Counselling Message” about drug-drug interactions between ART and the LNG implant, whereas 24 of the HIV-positive women included in our sub-study from Category C received only the”Post-Randomization Implant Counselling Message: about drug-drug interactions between ART and the implant. Finally, 3 HIV positive women who had been randomized to DMPA but discontinued it had all received the “Pre-Randomization Implant Counselling Message” about drug-drug interactions between ART and the LNG implant. These three women were included to ensure that their views were represented given that there were fewer women who discontinued their method. All women received these counselling messages during consent process at enrollment and these messages were repeated during their follow up visits. From November 2016 to June 2017, a total of 41 women participated in IDIs and 6 FGDs. Twenty-one women participated in both IDIs and FGDs, nine women participated only in the IDIs, and 11 participated only in the FGDs.

Women were told about the sub-study during their monthly main study follow-up visits, and those willing to participate in the IDIs were interviewed on the same day or at a later date of their choosing. There were approximately 18–22 women eligible for each Category (60 women total). To ensure that a full range of themes and issues were captured, up to 2 FGDs of 4–7 women were conducted for each Category (totalling up to 6 FGDs). Eligible women for the sub-study were recruited by a Research Assistant (JC) who was not previously involved with study activities. The Research Assistant contacted the eligible participants (either by their contact information provided to the study clinic or at their study visit).

We obtained consent from all participants interested in participating in the sub-study prior to their participation in the IDI or FGD. We conducted IDIs before the FGDs. We asked all IDI participants if they wished to take part in a FGD, and recruited additional women to participate in the FGDs to explore issues that emerged from the individual interviews in a group setting (FGD) to ensure data saturation. Our sample was drawn from participants in the RCT that met specific criteria based on the counselling they received (see below). Given the limited number of potential participants and the in-depth and open-ended interviewing approach, we felt it appropriate to offer participants the option to participate in both the IDIs and FGDs.

We used two semi-structured interview guides during the IDIs (Additional file 1: Appendix A) and FGDs (Additional file 2: Appendix B) which focused on participants’ understanding of counselling messages about potential risks associated with progestin-only contraception and HIV and how this understanding may have affected their decision discontinue their assigned contraceptive. Both IDI and FGD interview guides included questions about HIV knowledge and family planning use and factors affecting family planning. We present the results of an analysis of this data focusing on the knowledge, beliefs, and motivations to use contraceptive methods. Each FGD had a facilitator and note-taker, with these roles alternating between AB and JC. All IDIs and FGDs were conducted in Chichewa and audio-recorded with participants’ permission. Interviewers wrote summary notes immediately after the FGDs and IDIs to document key findings and then shared the notes with two other investigators (JT, KHT). The research team reviewed the summary notes after each interview and made necessary changes to the interview guides to capture any emerging topics as needed. The IDIs lasted about 25–30 min and the FGD lasted approximately 2 h.

### Data management and analysis

AB and JC simultaneously translated and transcribed IDI and FGD audiotapes into English. All transcripts were reviewed by four of the authors (AB, JC, KHT) for translation clarifications. Two team members (AB and KHT) developed deductive and inductive codes using a priori themes based on key domains from the interview guides and emerging themes identified during transcript review. The 4 emerging themes discussed in this manuscript include: (1) Knowledge about HIV transmission and source of infection; (2) Condom effectiveness for HIV and pregnancy prevention; (3) Motivating factors to use contraception; 4) Motivating factors for condom use. Table 1 outlines the major themes and sub-themes identified for this manuscript. Two coders (AB and KHT) double-coded all IDI and FGD transcripts using NVivo® 11 and met monthly to reconcile all code application and text segmentation. Coders used an iterative process to refine the codebook, adding new codes to incorporate emerging themes identified during the coding process and changing existing codes as needed. Once the codebook was finalized, all transcripts coded with previous versions were recoded using the final codebook. Coders indexed themes by code and participant characteristics using NVivo® 11. Coders used Microsoft Excel to and constructed matrices to summarize themes and collate them by participant characteristics. Results from the IDI and FGD analyses are presented interchangeably below. Findings exclusive to one interview type are noted as appropriate.

**Table 1:** Key themes and sub-themes

Theme	Sub-theme
1. Knowledge about HIV transmission and source of infection	A. HIV transmission
	1. Unprotected sex
	2. Other iatrogenic (razor blades, needles, blood transfusion)
	3. Maternal-to-child transmission
	B. Source of HIV information
	1. Health facilities
	2. Radio broadcasts and interpersonal contacts
	3. Study nurses
	C. Source of HIV infection
	1. Unfaithful partners 2. Partners reluctant to disclose
2. Beliefs about condom effectiveness for HIV and pregnancy prevention	1. Condoms prevent HIV, STIs, and pregnancy
	2. Worry about condom failure (e.g. breaking or having holes) for women without HIV
	3. Partners view condoms as ineffective
	4. Female condoms are more effective than male condoms due to female-control
3. Motivations for contraception	A. Facilitators
	1. Desire to improve economic status (with fewer children)
	2. Desire to focus on other priorities in life (with fewer children)
	3. Protection against pregnancy complications, such as blood loss
	4. Partner support
	5. Determination to use even without partner support (e.g. covert use)
	B. Facilitators—Women with HIV
	1. Pregnancy may lead to HIV progression, since both pregnancy and HIV suppress the immune system
4. Motivations for condom use	A. Facilitators
	1. Uncertainty about partner status
	B. Barriers
	1. Lack of woman’s control over decision making
	2. Situational use only (e.g. during menses or not using other contraceptive method)
	C. Facilitators—Women with HIV
	1. Avoid infection with drug-resistant HIV strain
	2. Dual protection for implant users due to drug interactions between ARV and implant

### Ethical considerations

This study was approved by the Institutional Review Boards of the National Health Sciences Research Committee of Malawi, the University of North Carolina School of Medicine, the US Centers for Disease Control and Prevention, and the Malawi Pharmacy, Medicines and Poisons Board. All women participated in an informed consent process that included written consent in Chichewa.

## Results

For both IDIs and FGDs, the mean age of women was 33.4 years, 30 (73.2%) women were HIV-positive, and 11 (26.8%) were HIV-negative (Table 2). Twenty-eight (68.3%) women were married or referred to their partner as “husband”, 9 (21.9%) were separated or divorced, 3 (7.3%) were widowed and 1 (2.4%) never married. During the interview, 15 women (36.6%) said they knew their partners were HIV positive, 6 (14.6%) knew their partners were HIV negative, and of the women who knew their partners status only 2 were in HIV discordant relationships (woman HIV positive/man HIV negative, man HIV positive/woman HIV negative). Eleven (26.8%) women (4 HIV negative and 7 HIV positive) did not know the HIV status of their partners. There were no responses for 9 participants (21.9%) on knowledge about their partner’s HIV status.

**Table 2:** Demographic and other characteristics of study participants

Characteristic	N = 41
Age (mean, SD)	33.4 (6.06)
Education (number, %)	
None	5 (12.20)
Some primary/primary	19 (46.34)
Some secondary/secondary	17 (41.46)
Number of children (mean, SD)	2.7 (1.11)
Relationship status (number, %)	
Married or partner called “husband”	28 (68.29)
Separated or divorced	9 (21.95)
Widowed	3 (7.32)
Never married	1 (2.44)
HIV status (number, %)	
Positive	30 (73.17)
Negative	11 (26.83)
Number of additional children desired (number, %)	
0	24 (58.54)
1–2	14 (34.15)
3–4	3 (7.32)
Roof material (number, %)	
None or grass	12 (29.27)
Metal, wood or cement	29 (70.73)
Trouble paying for food, clothing, medicine in past 12 months (number, %)	
Yes	15 (36.59)
No	26 (63.41)

### Knowledge about HIV transmission and source of infection

All participants (N = 41) talked about unprotected sex as a major mode of HIV transmission. Other reported modes of transmission were sharing razor blades, blood-to-blood contact, blood transfusion, sharing of unsterilized needles, breast milk, and maternal-to-child transmission.

I: Okay, how do you think a person gets HIV?

R: Through unprotected sex. Some say that through needles or razor blades, but the common way people get HIV is through unprotected sex. (IDI, HIV-positive)

Participants named a number of formal sources of HIV information including medical clinics, radio stations, and churches. The women also learned about HIV through family, friends and other informal community contacts. Most women considered medical institutions to be the main and most trusted source of HIV information primarily because they believed that health care workers were experts on HIV and family planning issues.

F: Who do you think is the most trustworthy source of information?

R: I think the hospital is the best place where you can get information. (FGD4, HIV-positive)

R: The hospital is where you can get the right information. These other people get their information from the hospital and broadcast on the radio. (FGD1, HIV-positive)

Given that the women received counseling messages in the main study about HIV acquisition and transmission risk with DMPA injection or LNG implant use, not surprisingly, some women named study nurses as their source of knowledge on this topic.

I had a feeling that once I use this method [LNG implant] I will not contract HIV but later on the nurses explained to me that the method does not prevent the risks of contracting HIV. That’s why I was concerned. But now I am no longer concerned because if I find a new sexual partner I will make sure to always use condoms. (IDI, HIV-negative)

Most HIV-positive women believed they contracted HIV through sexual contact with their male partners who had sexual relationships with other partners. They based this belief on becoming infected despite their faithfulness to their partner.

I just feel that I got infected from him because of his behaviour. I was faithful to him but he was promiscuous. (IDI, HIV-positive)

I got HIV from my husband. He is a driver and I saw him bringing other women to our house. It reached to the extent that I knew all his girlfriends. He had girlfriends in different bars and I knew all about that (FGD1, HIV-positive)

Another theme was that the women’s partners knew they were HIV positive but did not disclose their status to the women possibly due to fear of being blamed as bringing infection in the house. Some women talked about getting diagnosed while pregnant at the antenatal clinic, and when they disclosed their status to their partner, they discovered that he already knew he was infected and was probably waiting for this opportunity for the woman to have a test and put a blame on her.

I: How do you think you contracted HIV?

R: I think I got infected with HIV the time I was marrying my husband. By the time I came to the hospital when I was sick, he didn’t tell me that he was already HIV positive. He knew his status already and his immune system was okay during that time. (IDI, HIV-positive)

Another woman said her husband did not disclose that he was HIV positive until after they were tested for HIV together:

After the test, that’s when we were found to be HIV positive, and that’s when he told me that he is already on ART. By the time we went for the test we were not married, and he showed me his medication. (IDI, HIV-positive)

### Beliefs about condom effectiveness for HIV and pregnancy prevention

Most women regardless of HIV status understood the HIV and pregnancy risks associated with unprotected sex and believed that both male and female condoms, when used consistently, were effective protection against HIV, sexually transmitted infections (STIs), and pregnancy.

When you use condoms, the semen remains in the condom. If you are HIV positive there is no possibility of the virus from the man entering your body. (IDI, HIV-positive) Condoms are effective in many ways, like they can protect against sexually transmitted infections and pregnancy. (FGD5, HIV-negative)

Some HIV-negative women were worried that condoms provided less than 100% protection and therefore were ineffective in preventing HIV transmission. Skepticism about condom effectiveness emerged in two ways. First, some thought that condoms could slip or break if not used correctly.

I: Do you think condoms are effective at preventing HIV?

R: No

I: Why are they not effective?

R: A condom is not 100% effective because it can break.” (IDI, HIV-negative)

Second, other women talked about condoms with holes that put them at risk of contracting HIV or becoming pregnant:

Some people say that condoms have a hole in them while others are okay. So I don’t know the whole truth of it. (FGD2, HIV-positive)

For some women, concerns about condom effectiveness were reinforced by partners who also believed that condoms were not 100% effective in preventing HIV and pregnancy. This could negatively affect condom use among couples since men are most often the main decision makers about condom use.

I: Do you think your husband believes that condoms are effective at preventing HIV?

R: He does, but he also believes that they are not 100% effective.

I: Why does he think that way?

R: It can break anytime, it’s plastic! (IDI, HIV-negative)

Despite the fact that female condoms are not widely used by women in Malawi, several HIV-positive and negative women felt that female condoms were more effective protection than male condoms. One woman felt assured that a female condom was less likely to have been tampered with because she had control of it herself. As a result, these women felt it was important to encourage other women to use female condoms.

When they use female condoms, they can be assured of protection because with the male condom, you can see the man wearing it, but they can pierce it, and you might end up being pregnant or getting HIV. So I think the female condom is more reliable because you put it on yourself (FGD1, HIV-positive)

### Motivations for contraception use

The primary motivating factor for contraceptive use was a desire for fewer children to improve familial economic status and maternal and child health. Most women, regardless of HIV status, desired fewer children to lessen the economic hardships they face, especially when they were not in a stable relationship.

I have made up my mind not to have another child because he doesn’t help the children. I have been taking care of them alone. He takes advantage of me because I am working and I am able to support myself and the children, even pay school fees for them. (IDI, HIV-positive)

Contraceptive use was seen as a tool for controlling the number of children:

He came back to me in 2012 claiming that he wanted to take care of our second born child. By then our first born was 6 years old. Then I became pregnant again and that’s when he left me. So I don’t want to face the same problem. I decided to come for family planning because I feel he can come back again … I don’t want to become pregnant again. (IDI, HIV-negative)

Some women felt that contraception helped them plan for future pregnancies when they would have enough time to care for them:

I started using family planning because when you have children often, you do not have time to do other things and develop yourself and your home. However, when you use family planning, you are able to plan when to have children and develop yourself. There are distractions that come with children or pregnancy. (IDI, HIV-negative)

Women regardless of HIV status were aware of the problems that can arise during pregnancy, labor and delivery. Some talked about how women lose a lot of blood during labor and delivery, and therefore, women need to protect their health through contraception.

Some women have problems when giving birth. Others lose a lot of blood during delivery and require blood transfusion. So many of them think about their health when choosing family planning methods. They would like to prevent these problems so they chose to use family planning methods (FGD2, HIV-positive)

Childbirth experiences especially labour and delivery pain and post-partum complications (e.g. hemorrhage) were strong motivation for some women regardless of the number of children they had.

Like in my case, I have decided to come here to start using family planning methods because of what I faced during the time I was giving birth. ….I lost a lot of blood that required transfusion and I have made up my mind not to have another child despite having one child who is 15 years old. (FGD3, HIV-positive)

HIV-positive women talked more about protecting maternal health through contraceptive use despite the fact that many women felt that HIV positive women also had the right to have children. Nearly all HIV-positive women considered contraception important since both pregnancy and HIV are physically taxing and associated with immune suppression.

Like myself, I don’t care whether I will have an HIV negative baby or not, I think about my health and I know that if I become pregnant my body immunity may decrease or increase. …. So it’s better I should be using family planning methods. ….When you have children more often, your immunity decreases and you get sick often and your body doesn’t look healthy. (FGD1, HIV-positive)

When women give birth, some of them are weak and they decide to start using family planning. In addition to that, because of HIV many women decide to use family planning methods because when they have many children their body immunity drops and the fear of developing AIDS is one factor that encourages many women to use family planning. (FGD1, HIV-positive)

Additionally, HIV-negative women acknowledged the rights of HIV-positive women to have children but also encouraged them to think about their own health.

Women who are HIV positive have the right to have children but they need to think about their own health, the strength they had before they were infected and the strength they have now. They may have children but their immunity may drop more than a woman who is HIV negative. (FGD5, HIV-negative)

Both HIV-positive and negative women talked about the importance of discussing family size and contraceptive use with their partners. Women from both IDIs and FGDs acknowledged discussing with their partners plans to start family planning and that their partners’ approval played a positive role in their decisions to a mutually agreed upon contraceptive method.

In my case, I talked with my husband, and he said that we should be using family planning methods instead of having many children. When you have children often your immunity drops and you are not healthy; and also you cannot do many things because of the children. (FGD4, HIV-positive)

I was one of the people who used to have children every year, but when I sat down with my husband and told him that I would like to start using family planning methods he just asked me where I want to access the services. ….I started using family planning and my last born is 9 years old. (FGD6, HIV-negative)

One woman talked about the encouragement she received from her partner to use family planning in spite of her disinterest in doing so:

He thinks it is a good idea, I did not want to use family planning, he is the one who told me about it and convinced me to be using family planning. He said, “When you are using family planning, you have time to take care of the home and have plans to develop the family and ensure that the family has a good life.” (IDI, HIV negative)

Despite general agreement that partners should be involved in contraceptive use decisions, some focus group and in-depth interview participants preferred to make informed decisions on contraceptive use on their own. Many of these (women ultimately, used contraceptive methods covertly due to the lack of partner support. Despite their desire to make informed decisions about contraceptive use and the decision to use contraceptives covertly, they also acknowledged that covert use comes at a cost, especially if the partner discovered that the wife is using contraceptives without their knowledge or if side effects occur.

R: I made the decision on my own I didn’t consult him because I knew that if I did, he wouldn’t allow me to [use it]. I told him when I had already started using Depo, and he complained that I had wronged him because he wanted to have another child. (FGD4, HIV-positive)

Before his child was born I was on family planning. I used pills, so at the time I had two health passports, the one for family planning and the other one for day-to-day health-related problems. So I hid the other book and kept the other one in the open. (IDI, HIV-positive)

### Motivations for condom use

Several HIV-negative women talked about using condoms for HIV prevention. This was a particular concern among women who did not know their partner’s HIV status. The majority of these women expressed the concern that they could not accurately say if their partner was HIV negative or if he had ever been tested. They believed the best way to protect themselves from HIV was to always use condoms.

They told me that if I have unprotected sex, I may get HIV. I know my status because I got tested, but I do not know about my husband, maybe he is HIV positive. So I need to protect myself by using condoms. (IDI, HIV-negative)

In addition, some HIV-negative women said they always have condoms with them to be prepared for sex because sex often happens without any discussion about pregnancy, STI, or HIV prevention measures. Being prepared helps, but as one woman explained, it cannot protect you from all HIV risk since unprotected sex can happen unexpectedly.

Sometimes you might be sleeping and just realize that the man is on top of you [having sex]. (IDI, HIV-negative)

While some HIV-negative women were concerned about their risk for contracting HIV and STIs, others reported using condoms only during menses or when they were not using other contraceptive methods.

I was having my monthly periods and he really wanted to have sex with me. So to compromise, I told him to use a condom and he did. (IDI, HIV-negative)

I only used condoms while waiting for the date I was given to come back to the clinic to start family planning methods.” (IDI, HIV-negative)

On the other hand, condom use was considered very important by many HIV-positive women to avoid infection with a drug-resistant HIV strain and keep their HIV viral load low.

When both partners are taking ARVs [antiretroviral therapy], you may not fully trust your partner on whether they are taking the ARVs daily or not. If you do not use condoms, you share viruses that have developed resistance and that decreases your body immunity. (FGD1, HIV-positive)

The time we came for HIV testing, we were counselled that we should always use condoms to prevent infecting others and re-infecting ourselves. We were told that re-infection can reduce our immunity. That’s why he does not have any problems using condoms. (IDI, HIV-positive)

Most women said they had heard about the risks of becoming pregnant while taking ART and using the LNG implant from the study clinic for the first time. Some expressed concern that women in the community did not know about these messages and chose family planning methods (the LNG implant) that interfere with the efavirenz-based ART regimen, leading to unwanted pregnancies.

I heard the messages when I joined the study, before that people in the villages, they don’t know about these messages [drug-drug interaction between Efavirenz-based ART regimen and LNG implant]. Health workers come to our community, and just give out the birth control methods, and they don’t explain anything to the people. They don’t tell them anything about the drugs, even the fact that they can get pregnant. (FGD3, HIV-positive)

Some HIV-positive women expressed some concerns about the potential risks of becoming pregnant while taking Efavirenz based ART (5A) and using the LNG implant due to the counselling messages they received at each study visit about these risks. Most of them mentioned the importance of using condoms as counselled by the study nurses despite being on contraceptives to prevent pregnancy and their strong desire to always use them.

You should understand that it doesn’t mean that Jadelle^®^^2^ is 100% for people like me [people who are HIV-positive]. You can still get pregnant, that’s why we use condoms. (IDI, HIV-positive)

They told me that I should use condoms all the time because ARVs are more powerful than Jadelle^®1^, and if I don’t use condoms I may end up being pregnant. So they told me to use condoms all the time. (FGD3, HIV-positive)

One woman reported to have heard about the message about drug-drug interaction before joining the study and the counselling messages increased her desire to use condoms.

When they put the implant, they called me some time to come back to the hospital. They had made a mistake, they wrote different ARV drugs to the ones I was taking at the time. They told me that if I take that type of ARV drug with the one I am taking now I can easily get pregnant because the drug interferes with the Implant. That’s why we are encouraged to always use condoms. (FGD2, HIV-positive)

Nearly all HIV-positive women voiced that their partners theoretically agreed to condom use, but few used them consistently despite their effort to always have condoms with them and reminding their partners to use the. This could possibly be because most HIV-positive women either knew or suspected their partners were HIV-positive.

My husband accepts to use condoms most of the time, but there are times we don’t use them. I always put some condoms under the pillow, but there are times when I forget to put some, and I find it difficult to wake up and get them when we want to have sex because my husband insists that we should just have sex without a condom. (IDI, HIV-positive)

Women also talked about the difficulties they faced when requesting their partners use condoms. Some felt powerless to demand condom use while those who did were accused of having outside relationships or being promiscuous. Another challenge raised by some women concerned the negative stereotype about condoms and sex work. These women talked about how men incorrectly believe that condoms should only be used with female sex workers and not married women.

Well, they could give me the condoms, and when I go home and ask my husband to use them he would say, “Why should we use condoms? You must be a sex worker then because the only people I can use condoms with are sex workers because they are not safe. I can’t have sex using condoms with my wife, no!” (IDI, HIV-negative)

Other women talked about condom use side effects including how their partners found sex with condoms less pleasurable or fraught with physical side effects including the potential to develop a rash.

At first, he was complaining that sex with a condom is not pleasurable but now he is used to them and we always use condoms. (IDI, HIV-positive)

He is not comfortable using condoms because he has heard that people develop a rash when they use them. (IDI, HIV-positive)

## Discussion

Our findings showed that both HIV-positive and negative women were aware that HIV can be transmitted through unprotected sex and that only condoms when used correctly and consistently prevent HIV. The level of HIV knowledge of the women in this study is consistent with findings from the 2015–2016 Malawi Demographic and Health Survey, which demonstrated that both women and men had notable levels of HIV knowledge about modes of transmission and prevention [13]. Our study showed that women considered health institutions to be their primary trusted source of HIV information despite the fact that they also learned health information from other trusted community sources, including family, friends and religious organizations. In one study conducted in Malawi and another in Cameroon, researchers also found that health personnel were the primary trusted source of information about contraceptives [16, 17].

Childbearing is a key identity and social status factor for women and men in Africa. In other studies, fertility intentions of HIV-positive women increased because of their ART use and the consideration of child bearing as an important part of marriage and social identity [18–20]. HIV-positive women in this study were motivated to use contraceptive methods despite being on ART, to limit family size. Their motivation to do so was in part to protect their health due to personal experience with complications during labour and delivery but also because they believed that becoming pregnant while HIV positive can affect their immunity. While pregnancy has not been associated with HIV disease progression, particularly in the era of ART [21], pregnancy and HIV infection are both associated with immune suppression [22], which may lead women to think that pregnancy would affect their immunity.

Our findings also showed that the main study counselling messages about drug-drug interactions between ART and hormonal contraceptives motivated HIV-positive women to use condoms when having sex with their partners. However, women mentioned several reasons that made it difficult for them to use condoms consistently with their partners as desired. First, for both HIV-positive and negative women, male partner refusal was more due to individual level or relationship dynamics than lack of knowledge. Results suggest that HIV-negative women were more concerned about male partner’s refusal to use condoms due to their uncertainly about the HIV status of their partners. On the other hand, HIV-positive women were worried about partner refusal because of concerns over infection with drug-resistant HIV strains and keeping their viral counts low. The challenge to use condoms among HIV positive women in this study due to partner’s opposition is consistent with findings by Haddad et al. (2018) which found that ART and contraceptive use did not deter condom use among HIV-positive individuals in Malawi and that how condom use was likely due to individual and relationship factors [23]. Secondly, women expressed a concern over negative stereotypes that characterize women who use condoms as promiscuous or as sex-workers, to the extent that condom use may be more acceptable in extramarital affairs. This is supported by other studies indicating that condoms use among married couples implies that one of the partners has extra marital affair or suspected the other partner of outside sexual relationships [24, 25]. Negative stereotypes impair willingness of both women and men to use condoms, hence the need to educate couples on the importance of using condoms and improving couple communication about condom use.

Many women talked about men’s reticence to use condoms inside committed relationships including marriage. This reluctance was based on the belief that condoms should be used only in extramarital partnerships to prevent STIs and HIV transmission from secondary to primary partners. Although consistent and correct condom use by men with other partners might offer additional protection to women, this practice further complicates an already fraught condom negotiation process by putting much of the woman’s infection risk into the hands of her partner. Considering the high rates of HIV among women of childbearing age in Malawi [13], it is unclear to what extent this belief is actually put into practice by men with extramarital relationships. In this study, men’s refusal to use condoms stemmed largely from concerns about reduced sexual pleasure and side effects. Previous studies in Malawi and Sub-Saharan African countries have found that men who believe that condoms decrease pleasure are less likely to use them [26–28]. Finally, access to female condoms for women in this study (and for women generally in Malawi) is limited, making condom use more challenging because male condoms require partner cooperation [29]. Therefore, we recommend improved access for women to condoms in all health facilities in Malawi. Importantly, we found that some women believed that female condoms were more effective at preventing pregnancy or STI/HIV than male condoms. Preference for female condoms was expressed due to male partners’ refusal to use male condoms. Gendered concerns remain a significant challenge to condom use in Malawi and other SSA countries and may require new technologies to prevent HIV transmission, including the vaginal ring containing drugs that protect against HIV [30, 31], pre-exposure prophylaxis (PrEP) [32–35] and HIV vaccine which are female controlled if and when they become available in the country [36].

We found that women who consulted and made contraceptive decisions with their spouses were more likely to continue using their contraceptive without interference, unlike those who made the decision alone. Previous studies have also demonstrated this relationship [37–39]. In one study conducted in Luanda, Angola, perceived partner’s approval was positively associated with contraceptive use [39]. Similarly, a study in Nigeria and Mozambique found that men's awareness of and support for use of contraceptives were markedly associated with their spouses' desire to use contraception [37, 38]. The women in both IDIs and FGDs who suspected partner disapproval said that they used contraception without their partners’ knowledge. These women’s decisions to use contraception rather than conforming to social norms or expectations is notable. A cross-sectional study conducted in Ghana found husband’s opposition to be the major reason women did not access family planning services [40].

Our findings have important implications for programming efforts aimed at improving contraceptive and condom use among both HIV positive and negative women. Our findings indicated that women who consulted and made contraceptive decisions with their spouses were more likely to continue using their contraceptive without interference because of the support they received from their partners. Our findings further showed that both HIV-positive and negative women face challenges using condoms despite their knowledge through the counselling messages that progestin contraceptive methods (i.e., DMPA injection and LNG implant) would not protect them from contracting HIV and that the interaction of progestin hormonal contraception with efavirenz-based ART might increase the risks of pregnancy and HIV acquisition. This underscores the need for improved counselling messages on condom and contraceptive use for both women and their partners as part of comprehensive family planning programs for both HIV-positive and negative women. This calls for concerted efforts to strengthen male involvement in reproductive health services to promote their understanding of the importance of condom and hormonal contraceptive use and provision of couples’ counselling services should be considered to improve condom use. The challenge of reducing maternal and infant mortality in the SSA and in Malawi, might be better addressed with intervention strategies involving men, both to increase knowledge about contraceptive methods but also to engage more couples in cooperative decision making about condom and contraceptive use.

Since most of the participants who participated in our study are in a close relationship, our findings further point to areas where further studies are needed to further explore how the type of relationship of women affects contraceptive and condom with their partners. One other potential avenue would be to explore how existing condom use norms (with secondary/extramarital partners) might be used to protect primary partners from HIV and STIs.

A limitation of this study is that we recruited and interviewed women who participated in a randomized clinical trial where they were randomized to a progestin contraceptive so they were already intending and motivated to use hormonal contraception to prevent pregnancy. In addition, during the trial, they received counselling messages about the risks of DMPA and LNG implant on HIV acquisition and transmission, as well as messages about possible drug interactions between the LNG implant and ART. Therefore, participants may have been better informed about contraception and HIV compared to other Malawian women. For these reasons and as with other qualitative studies, considerations should be taken when generalizing results of this study.

## Conclusion

In this study, we found that both HIV positive and negative women were highly motivated to use family to limit family size and also to protect their own health because of the knowledge that frequent pregnancies can affect their health. The message about drug-drug interaction between effavirenz-based ART and family planning method did not affect family planning use among HIV positive women interviewed in this study. Intensive counselling about potential risks of family planning failure due to drug-drug interaction with Effavirenz based ART should therefore, be intensified to promote understanding among women on ART and the need for condom use. However, there seemed to be a challenge to use condoms without partner involvement despite the desire by most women in our study to use condoms. Effective contraceptive and condom use requires partner education and involvement in the decision–making process.

## Supplementary Information

**Additional file 1: Appendix A**. Qualitative sub-study interview guide.

**Additional file 2: Appendix B**. Focuss group discussion notetaking guide (English).

**Additional file 3: Appendix C**. Guideline checklist for reporting qualitative studies.

## Data Availability

Data available on request as other data analyses are in progress and release could prevent future publication of these analyses.

## Abbreviations

DMPA: Depo Medroxyprogesterone Acetate injectable
FGD: Focus group discussion
IDI: In-depth interview
SSA: Sub-Saharan Africa
UNC: University of North Carolina
ART: Antiretroviral therapy
STI: Sexually transmitted infections

## Footnotes

1 Evidence at the time suggested an interaction for HIV acquisition but that data now show this not to be the case according to the Evidence for Contraceptive Options and HIV Outcomes (ECHO) trial. https://www.medscape.com/viewarticle/914482

2 Use of trade names and commercial sources is for identification only and does not imply endorsement by the Centers for Disease Control and Prevention, the Public Health Service, or the U.S. Department of Health and Human Services.
