# Supplementary material for: Motivations to use hormonal contraceptive methods and condoms among HIV-positive and negative women randomized to a progestin contraceptive in Malawi: a qualitative study
Source: BMC Womens Health. 2021 Mar 20;21:114. doi: 10.1186/s12905-021-01236-1 (PMC7981805; doi:10.1186/s12905-021-01236-1)
Supplement: Supplementary file 1 — Additional file 1: Appendix A. Qualitative sub-study interview guide. [file 12905_2021_1236_MOESM1_ESM.doc]

**Appendix J: Qualitative Sub-Study Interview Guide**

**December 13, 2016**

**I. Focus Group Discussions**

- To focus on those exposed to different counseling messages

- Will divide groups so that some have HIV+ women only and others have HIV- women only

**Questions:**

Thank you all for joining us today for this discussion. You have all been invited to participate in this discussion because you have been participating in a study where you were randomized to receive either the injection or Jadelle. We wanted to better understand your thoughts about HIV and family planning and what your communities think about these issues. We’ll start out by asking some general questions about your community.

1. What do you think are the most important issues facing people in your community today?
2. There are a lot of things that people might be thinking about when it comes to family-related issues, like when to have another child, or maybe even whether to stop having children.
   1. What do most people in your community think about this?
   2. How many kids do most want and why?
   3. How do you think couples talk to each other about how many kids they want?
      1. *PROBE*: How does the use of family planning play a role in these conversations about spacing or limiting the number of children they have?
3. Another thing to think about, here in Malawi, is HIV.
   1. What do women think about their risk for HIV?
   2. How do they talk to their partners about HIV?
      1. *PROBE*: How they talk about HIV risk and ways to reduce HIV risk?

Next, we’d like to talk a bit more about how you and others in the community talk and think about family planning and HIV issues.

1. What have you all heard about family planning,

What do people in the community say about family planning? *PROBE:*

- 1. How acceptable is it to use family planning?
  2. What family planning methods have you heard about, and what have you heard about of these methods?
     1. *PROBE:* have you heard any good things about the methods?
     2. *PROBE:* have you heard any bad things about the methods?

1. From where or whom do you get your family planning information?
   1. Who do you think is a more trustworthy source for family planning information?
   2. Do people talk about different things with their friends than with their partners? Other family members? Providers? Why do you think they do that?
2. What do people say about how to prevent HIV?
   1. *PROBE*: In your opinion, do you think people think that condoms are effective at preventing HIV? What makes them think in that way?
3. What do people say about getting treatment with antiretroviral therapy?
   1. *PROBE:* What do people think are the risks or benefits of taking antiretroviral therapy? What makes them think in that way?
4. Who talks about these things/who do you hear them from?
   1. Is there anyone you think is a more trustworthy source for HIV information? Other family members? Providers? Why?
5. You might remember that during the study you have been in, the study nurses have been talking with you about Jadelle, pregnancy, and HIV-infected women who are taking HIV drugs.
   1. What do you remember about these discussions?
   2. Had you ever heard any of this information before? From where/who?
   3. Do you think other people in the community know this information? Where do you think they heard this information from?
   4. Did you talk to anyone in the community about this information after the study nurses talked to you about it?

*READ:* When you were consented to participate in this study, you were read the following message: *“*We do not know whether HIV drugs that you are taking or drugs you take in the future make the contraceptive implant less effective. There have been a few unexpected pregnancies in women with implants who were on HIV drugs. Therefore, you should also always use condoms to prevent pregnancy as back-up protection if you have an implant and you are on HIV drugs.”

1. What do you think this message is saying? *Answer any questions about the message and correct any misperceptions about it.*
2. Do you think knowing this (correct) information might make someone change their minds about using Jadelle?
   1. Who would change their minds?
   2. Under what conditions?
   3. Why would they change their minds?
   4. What might they do instead to help prevent pregnancy?
   5. What about people who decided to use Jadelle anyway, what do you think led them to stick with that decision?
   6. How do you think we could make the counseling script easier to understand?
3. You might also remember that during the study that you have been in, the study nurses have been talking to you about using hormonal contraception and HIV transmission.
   1. What do you remember about these discussion?
   2. Had you ever heard any of this information before? From where?
   3. Do you think other people in the community know about this information? Where do you think they heard this information from?
   4. Did you talk to anyone in the community about this information after the study talked to you about it?

*READ:* When you were consented to participate in this study, you were read the following message: *“*We do not know whether the birth control injection or implant may increase your risk of getting HIV or giving HIV to your partner. If you have HIV, neither the birth control injection nor implant will prevent you from giving HIV to your partner. If you do not have HIV, neither the birth control injection nor implant will prevent you from getting HIV from a partner who has HIV. Neither the birth control injection nor implant will protect you from sexually transmitted infections. Therefore, you should always use condoms to prevent HIV and sexually transmitted infections.”

1. What do you think this message is saying? *Answer any questions about the message and correct any misperceptions about it.*
2. Do you think knowing this correct information might make someone change their minds about using hormonal contraception? By hormonal contraception we mean either the birth control injection or implant.
   1. Who would change their minds?
   2. Under what conditions?
   3. Why would they change their minds?
   4. What might they do instead to help prevent pregnancy?
   5. What about people who decided to use hormonal contraception (either birth control injection or implant) anyway, what do you think led them to stick with that decision?
   6. How do you think we could make the counseling message easier to understand?

Thank you for your participation in this discussion today. This information will help us to design future educational materials to help providers talk to women about HIV and family planning. Please let us know if you have any questions. *[Clarify any misperceptions if any came up during the discussion.]*

**II. In-Depth Interviews**

- To focus on those who continued versus discontinued their assigned method.

- To explore the reasons why women were willing to participate in the study despite the possibility that their randomized contraceptive might increase HIV acquisition or transmission.

- To explore the reasons why women were willing to be randomized to Jadelle despite the possibility that it may be less effective for women taking HIV drugs.

- To explore reasons for condom use or non-use.

**Questions**

Thank you for agreeing to participate in this interview. You were invited for this interview because you have been participating in another study where you were randomized to receive either the injection or Jadelle. We wanted to better understand your thoughts about the family planning method you were randomized to and why you have chosen to continue or discontinue it. We’ll start out by asking some general questions about the reasons you use or do not use family planning. There are many women and couples who use family planning methods for a lot of different reasons.

1. What are the reasons you have chosen to use or not use family planning?
2. How long have you been with your most recent partner?
3. What does your partner think about family planning? PROBE:
   1. Does he know that you are using FP?
      1. *If yes, how (e.g. from whom, under what circumstances, when) did he find out?*
   2. Does he agree with you that you should be using FP? Why or why not?
      1. *What was his reaction to you using FP?*
   3. Does he agree with your reasons for using it? Why or why not?
   4. How many children do you have?
   5. How many children does your partner have?
   6. Did you talk to him about when to have a/another child?
      1. *What was his preference/opinion?*
   7. *If HIV+, does your partner know your HIV status?*
      1. *If yes, how did he react when you told him?*
      2. *How did you find out your HIV status? How long ago was this?*
      3. *Do you know how you got infected with HIV?*
      4. *When did you start taking ART?*
   8. *Do you know your partner’s HIV status?*

*If yes, what is his status?*

*If yes, how did he find out his HIV status? How long ago was this?*

1. What about your friends and family? Do they think family planning is a good or bad thing or neither? Why?
   1. (If they mention family/friends’ use of a specific method): *PROBE: Why did they choose to use (insert method if applicable)? Where do they access/get this method?*
   2. *PROBE: If you think about women generally, so not just your family or close friends, do you think that most women understand the importance of family planning? Or that most women understand the desire to limit family size?*
   3. *PROBE: Do you think that most men feel the same way? Why or why not?*
   4. *PROBE:* Do your friends and family think that women who are HIV-infected should use different methods than women who are not HIV infected? *Why or why not?*
2. When you decided to join this study did you have any concerns about having the study nurses assign you to a birth control method rather than choosing it yourself? Why or why not?
3. What was the method of birth control that the study nurses assigned to you?
   1. How did you like that method? Were there things about it that you didn’t like?
   2. Had you ever used this method in the past (before you joined this study)?
   3. How did you like it then?
4. What method of birth control are you using now?
5. *For those who continued original study method:*
   1. Why did you decide to keep using that method?
      1. *PROBE*: Did someone encourage you to continue to use this method? Who. . . partner, family, friend, study nurses, other influences?
      2. *PROBE*: What did they say about the method? Was this something they experienced or did they hear that through word of mouth?
6. *For those who switched to a different method or no method:*
   1. Why did you decide to stop using your original method?
      1. *PROBE*: *What specific concerns or side effects did you have about the method?*
      2. Did someone encourage you to discontinue to use this method? Who . . . partner, family, friend, other influences?
      3. *PROBE*: What did they say about the method? Was this something they experienced or did they hear that through word of mouth?
   2. *For those who had their Jadelle removed*:
      1. *Did you have the Jadelle removed in our study clinic*? Why or why not?
   3. *For those of you using a different method:*
      1. *What things do you like about the new method?*
      2. *What are the things that you do not like about the new method?*
7. What are the things that are most important to you when you are choosing a method of birth control?
   1. How important is the effectiveness of the method to you? Or how well it prevents pregnancy?
   2. How important is the convenience of the method? Or how long the method lasts before you need to go to the clinic to get more?
   3. How important are the side effects of the method? Or how it affects you physically?
      1. *PROBE: Is it important to you to have monthly periods while using the method? Why or why not?*
   4. How important is your ability to hide it/keep it a secret from your partner? Why or why not??
8. How important are the opinions of other people when you are choosing a method of birth control?

a. PROBE: How important are the opinions of your partner, family, friends, other influences?

1. How do you think a person gets HIV?

a. From where/whom did you learn this information?

As part of this study, the study nurses talked to you about family planning methods, especially Depo and Jadelle.

1. Can you tell me what you remember about what they told you about whether using Depo and Jadelle could increase the chance that an HIV-negative woman will get HIV if her partner is infected with HIV?
2. Can you tell me what you remember about what they told you about whether using Depo and Jadelle could increase the chance that an HIV-positive woman will give HIV to her partner/husband if he is HIV-negative?
3. Had you heard either of these messages before? Where/from whom?
   1. *PROBE: If radio broadcast or other media is mentioned: Which station? How long ago?*
4. Can you tell me whether these messages were a concern for you when you agreed to join the study?
   1. Why or why not?
   2. How much of a concern are the messages to you now?
   3. Did the messages increase your use of condoms? Why or why not?
   4. Many women have challenges with using condoms because their partners refuse to use it or they forget. How often do you use condoms in your current relationship?
      1. *PROBE: How difficult is it for you to use condoms in your relationship?*
      2. *PROBE: Does your partner ever refuse to use condoms? If so, how often does he refuse?*
      3. *PROBE: Are there any other reasons why you do or do not use condoms regularly?*
      4. *PROBE: Do you think that condoms are effective at preventing HIV? Why or why not?*
      5. *PROBE: How does your husband/partner feel about condoms? Does he think they are effective at preventing HIV?*
5. Can you tell me what you remember about what the study nurses told you about Jadelle and whether taking antiretroviral medicine can make it less effective at preventing pregnancy?
   1. Is that something you had heard before? Where/from whom?

*If woman is HIV-negative, Skip to last paragraph of Interview guide.*

1. *HIV- positive women ONLY:*
   1. Can you tell me whether this message was a concern when you agreed to join the study? Why/ why not?
   2. How much of a concern is it to you now?
   3. Did it increase your use of condoms? Why or why not?
      1. *PROBE:* Do you think that condoms are effective at preventing pregnancy?
      2. *PROBE:* Does your husband/partner think that condoms are effective at preventing pregnancy?

*If woman is not on implant and ART, Skip to last paragraph of interview guide.*

*For HIV-positive women on implant and ART ONLY, READ:* At the end of every Physical Exam during the Study Extension, you are read the following message: *“*Please also remember that we do not know if the HIV drugs that you are taking make the contraceptive implant less effective. You should also always use condoms to prevent pregnancy as back-up protection if you have an implant and you are on HIV drugs.”

1. What do you think this message is saying?
2. Do you have any questions about this information?
   1. Does this information raise any concerns about using Jadelle?

*Note to interviewer: If participant has concerns and they are on a method they are concerned about, refer them to a nurse who can give them more in-depth counseling and help them switch methods if that is what they desire.*

Thank you for your participation in this discussion today. This information will help us to design future educational materials to help providers talk to women about HIV and family planning. Please let us know if you have any questions. *[Clarify misperceptions if any came up during the interview, and ask them if they have any additional questions or concerns about the correct information. If they have concerns about a method that they are using, refer them to the study nurse to give them more in-depth counseling and help them to switch methods if that is what they desire.]*
