# Supplementary material for: Motivations to use hormonal contraceptive methods and condoms among HIV-positive and negative women randomized to a progestin contraceptive in Malawi: a qualitative study
Source: BMC Womens Health. 2021 Mar 20;21:114. doi: 10.1186/s12905-021-01236-1 (PMC7981805; doi:10.1186/s12905-021-01236-1)
Supplement: Supplementary file 2 — Additional file 2: Appendix B. Focuss group discussion notetaking guide (English). [file 12905_2021_1236_MOESM2_ESM.doc]

**Focus Group Discussion Notetaking Guide (English)**

- To focus on those exposed to different counseling messages

- Will divide groups so that some have HIV+ women only and others have HIV- women only

**Category (A/B/C): _______ FGD #: ________ # of FGD participants: __________**

**Randomized method (Depo/Jadelle): _____________**

**Date (DD/MMM/YY): _____________________ Time FGD Started (HH:MM): _________________**

**Questions:**

Thank you all for joining us today for this discussion. You have all been invited to participate in this discussion because you have been participating in a study where you were randomized to receive either the injection or Jadelle. We wanted to better understand your thoughts about HIV and family planning and what your communities think about these issues. We’ll start out by asking some general questions about your community.

1. What do you think are the most important family planning issues facing people in your community today?
2. There are a lot of things that people might be thinking about when it comes to family-related issues, like when to have another child, or maybe even whether to stop having children.
   1. What do most people in your community think about this?
   2. How many kids do most want and why?
   3. What do couples say to each other when they talk about how many kids they want?
      1. *PROBE*: How does the use of family planning play a role in these conversations about spacing or limiting the number of children they have?

d.    There are many reasons why women decide to use family planning. To what extent do women decide to get on family planning to protect their own health?

                                          i.    *PROBE*: What are some of the reasons women may want to protect their health? (e.g. loss of blood during pregnancy, childbirth and weakness, “low immunity” and frequent childbirth)

1. Protecting health is one of the things that doctors and nurses try to do for their patients. You may have heard your doctor or nurse talk about the “risk” of getting sick or the “risk” of getting pregnant.
   1. When doctors and nurses talk about “risk” like that, what do they mean? What are they talking about?
      1. PROBE: Can someone give me an example of risky behavior that might lead to getting sick? What about risky behavior that might lead someone to getting pregnant (who doesn’t want to be pregnant)?
      2. PROBE: Do you think most people make decisions to prevent sickness or getting pregnant because they are worried about “risk”? OR Do most people not think about “risk” before the act?
2. Another thing to think about, here in Malawi, is HIV.
   1. What do women think about their risk for HIV?
      1. PROBE: Do most women think that they are at risk for HIV?
   2. What do they say when they talk to their partners about HIV?
      1. *PROBE*: What do they say when they talk about HIV risk and ways to reduce HIV risk?

Next, we’d like to talk a bit more about how you and others in the community talk and think about family planning and HIV issues.

1. What have you all heard about family planning,

What do people in the community say about family planning? *PROBE:*

- 1. How acceptable is it to use family planning?
  2. In your opinion, do you think people in the community think that HIV positive women should have children? Why or why not?
  3. Early we talked about how some women may want to use family planning to protect their health. [Mention some of the issues raised]. How are these things related to HIV status? Do women who are HIV positive worry more about these things in relation to their health?
  4. What family planning methods have you heard about?
     1. *PROBE:* What good things have you heard about these methods?
     2. *PROBE:* What bad things have you heard about these method?
     3. Have you heard of any traditional methods of birth control? *PROBE: Have you heard about the string method?*
        1. If so, how do you think it works, and how effective do you think it is?

1. From where or whom do you get your family planning information?
   1. Who do you think is a more trustworthy source for family planning information?
   2. Why do you think they are the most trustworthy source?
   3. Do people talk about different things with their friends than with their partners? Other family members? Providers? Why do you think they do that?
   4. Does your church or mosque ever give out family planning information? If so, what sort of information do they give? Which denomination do you belong to?
   5. There are several family planning methods such as pills, depo, Norplant, loop, condoms. How do women decide which of these methods to use?
      1. PROBE: Is it advice from someone else (friend, relative, partner, doctor), their own experience, or something about the method (characteristic/side effects)?
   6. What do you think women think is the most important characteristic that they want in their family planning method? [Note: let them generate a list first, then go to PROBE]
      1. PROBE: Is their effectiveness, or their side effects, or having regular menses, or being able to hide it from their partner, or something else?
2. What do people say about how to prevent HIV?
   1. *PROBE*: In your opinion, do you think people in your community think that condoms are effective at preventing HIV? What makes them think in that way?
3. What do people say about getting treatment with antiretroviral therapy?
   1. *PROBE:* What do people in your community think are the benefits of taking antiretroviral therapy?
   2. What do people in your community think are the risks of taking antiretroviral therapy?
   3. What makes them think in that way?
4. Who talks about these things/who do you hear them from?
   1. Who do you think is the most trustworthy source for HIV information? Other family members? Providers? Why?
5. You might remember that during the study you have been in, the study nurses have been talking with you about Jadelle, pregnancy, and HIV-infected women who are taking HIV drugs.
   1. What do you remember about these discussions?
   2. Had you ever heard any of this information before? From where/who?
   3. Do you think other people in the community know this information? Where do you think they heard this information from?
   4. Did you talk to anyone about this information after the study nurses talked to you about it?
      1. If Yes, who did you talk to about it, and why did you decide to talk to that person? Did you talk to your partner, friends, or other family members about it?
      2. What was their reaction when you talked to them about it?

*READ:* When you were consented to participate in this study, you were read the following message: *“*We do not know whether HIV drugs that you are taking or drugs you take in the future make the contraceptive implant less effective. There have been a few unexpected pregnancies in women with implants who were on HIV drugs. Therefore, you should also always use condoms to prevent pregnancy as back-up protection if you have an implant and you are on HIV drugs.”

1. What do you think this message is saying? *Answer any questions about the message and correct any misperceptions about it.*
   1. What do you think it means when it says that the birth control injection or implant MAY increase your risk?
   2. What is a good way to talk to people about a potential increase in risk without scaring them or thinking that the risk is definitely there?
2. Do you think knowing this (correct) information might make someone change their minds about using Jadelle?
   1. Who would change their minds?
   2. Under what conditions?
   3. Why would they change their minds?
   4. What might they do instead to help prevent pregnancy?
   5. What about people who decided to use Jadelle anyway, what do you think led them to stick with that decision?
   6. How do you think we could make the counseling script easier to understand?
3. You might also remember that during the study that you have been in, the study nurses have been talking to you about using hormonal contraception and HIV transmission.
   1. What do you remember about these discussion?
   2. Had you ever heard any of this information before? From where?
   3. Do you think other people in the community know about this information? Where do you think they heard this information from?
   4. Did you talk to anyone about this information after the study talked to you about it?
      1. If Yes, who did you talk to about it, and why did you decide to talk to that person? Did you talk to your partner, friends, or other family members about it?
      2. What was their reaction when you talked to them about it?

*READ:* When you were consented to participate in this study, you were read the following message: *“*We do not know whether the birth control injection or implant may increase your risk of getting HIV or giving HIV to your partner. If you have HIV, neither the birth control injection nor implant will prevent you from giving HIV to your partner. If you do not have HIV, neither the birth control injection nor implant will prevent you from getting HIV from a partner who has HIV. Neither the birth control injection nor implant will protect you from sexually transmitted infections. Therefore, you should always use condoms to prevent HIV and sexually transmitted infections.”

1. What do you think this message is saying? *Answer any questions about the message and correct any misperceptions about it.*
   1. What do you think it means when it says that the birth control injection or implant MAY increase your risk?
   2. What is a good way to talk to people about a potential increase in risk without scaring them or thinking that the risk is definitely there?
2. Do you think knowing this correct information might make someone change their minds about using hormonal contraception? By hormonal contraception we mean either the birth control injection or Jadelle.
   1. Who would change their minds?
   2. Under what conditions?
   3. Why would they change their minds?
   4. What might they do instead to help prevent pregnancy?
   5. What about people who decided to use hormonal contraception (either birth control injection or Jadelle) anyway, what do you think led them to stick with that decision?
   6. How do you think we could make the counseling message easier to understand?

Thank you for your participation in this discussion today. This information will help us to design future educational materials to help providers talk to women about HIV and family planning. Please let us know if you have any questions. *[Clarify any misperceptions if any came up during the discussion.]*

**Time FGD Ended: _________________** **3-digit Notetaker Code: ________**

**NOTES/COMMENTS ABOUT FOCUS GROUP DISCUSSION:**

1) General environment (interruptions, etc.):

2) Group disposition (attentive, distracted, mood, etc.):

3) Clinical issues and referrals:

4) Other:
