# Supplementary material for: Motivations to use hormonal contraceptive methods and condoms among HIV-positive and negative women randomized to a progestin contraceptive in Malawi: a qualitative study
Source: BMC Womens Health. 2021 Mar 20;21:114. doi: 10.1186/s12905-021-01236-1 (PMC7981805; doi:10.1186/s12905-021-01236-1)
Supplement: Supplementary file 3 — Additional file 3: Appendix C. Guideline checklist for reporting qualitative studies. [file 12905_2021_1236_MOESM3_ESM.doc]

**Guideline Checklist for reporting Qualitative studies**

BMWH-20-00228: Bula et al. “Motivations to use hormonal contraceptive methods and condoms among HIV-positive and negative women randomized to a progestin contraceptive in Malawi: A qualitative study.”

| Item | Guide questions/ description | Comment/Line number in manuscript |
| --- | --- | --- |
| Domain 1: Research team and reflexivity  Personal characteristics | 1. Interviewer/facilitator Which author/s conducted the interview or focus group? | Agatha Bula  John Chapola |
|  | 2. Credentials What were the researcher’s credentials? E.g. PhD, MD | Agatha Bula PhD  John Chapola MSc |
|  | 3. Occupation What was their occupation at the time of the study? | Agatha Bula Nurse/Midwife  John Chapola Social worker |
|  | 4. Gender Was the researcher male or female? | Agatha Bula Female  John Chapola Male |
|  | 5. Experience and training What experience or training did the researcher have? | Both were trained in qualitative data collection, coding and analysis |
| Relationship with participants | 6. Relationship established Was a relationship established prior to study commencement? | Both had no any relationship with the participants. They were both not involved in the main study |
|  | 7. Participant knowledge of the interviewer What did the participants know about the researcher? e.g. personal goals, reasons for doing the research | Participants did not know anything about the researchers. |
|  | 8. Interviewer characteristics What characteristics were reported about the interviewer/facilitator? e.g. Bias, assumptions, | None |
| Domain 2: study design  Theoretical framework | 9. Methodological orientation and Theory What methodological orientation was stated to underpin the study? e.g. grounded theory, discourse analysis, ethnography, phenomenology, content analysis | Content analysis |
| Participant selection | 10. Sampling How were participants selected? e.g. purposive, convenience, consecutive, snowball | Purposive sampling |
|  | 11. Method of approach How were participants approached? e.g. face-to-face, telephone, mail, email | Participant were approached Face-to-face when they came for their main study follow-up visits. A list of potential participants was given to main study staff to notify the qualitative team when the participant reported for her visit. |
|  | 12. Sample size How many participants were in the study? | 41 |
|  | 13. Non-participation How many people refused to participate or dropped out? Reasons? | None |
| Setting | 14. Setting of data collection Where was the data collected? e.g. home, clinic, workplace | IDIs were conducted at the Study Clinic and FGDs were conducted at a private conference room on the campus of Kamuzu Central Hospital in Lilongwe |
|  | 15. Presence of non-participants Was anyone else present besides the participants and researchers? | No |
|  | 16. Description of sample What are the important characteristics of the sample? e.g. demographic data, date | Women of child bearing age, both HIV positive and negative. |
| Data collection | 17. Interview guide Were questions, prompts, guides provided by the authors? Was it pilot tested? | Questions on the guides were provided by the authors and revised as needed during implementation in keeping with qualitative methods of data collection |
|  | 18. Repeat interviews Were repeat interviews carried out? If yes, how many? | 21 participants participated in both IDIs and FGDs. |
|  | 19. Audio/visual recording Did the research use audio or visual recording to collect the data? | Audio recording |
|  | 20. Field notes Were field notes made during and/or after the interview or focus group? | Yes |
|  | 21. Duration What was the duration of the interviews or focus group? | The IDIs lasted about 25-30 minutes and the FGD lasted approximately 2 hours. |
|  | 22. Data saturation Was data saturation discussed? | Women were recruited when they came for their monthly main study follow-up visits, so there was a limit to the number of women who could be recruited. Reaching saturation is more a concern in situations without potential recruitment caps. Priority was placed on ensuring that interview guides/interview process captured required topics in an in-depth and complete manner. |
|  | 23. Transcripts returned Were transcripts returned to participants for comment and/or correction? | No |
| Domain 3: analysis and findings  Data analysis | 24. Number of data coders How many data coders coded the data? | 2 |
|  | 25. Description of the coding tree Did authors provide a description of the coding tree? | Yes |
|  | 26. Derivation of themes Were themes identified in advance or derived from the data? | Themes were identified both in advance and derived from the data. |
|  | 27. Software What software, if applicable, was used to manage the data? | NVivo® 11 |
|  | 28. Participant checking Did participants provide feedback on the findings? | No |
| Reporting | 29. Quotations presented Were participant quotations presented to illustrate the themes / findings? Was each | Yes |
|  | quotation identified? e.g. participant number | Quotes were identified by the researchers prior to the final version of the manuscript to ensure participant diversity in quotes used as examples; quotes are now identified by method (IDI or FHD & HIV status) |
|  | 30. Data and findings consistent Was there consistency between the data presented and the findings? | Yes |
|  | 31. Clarity of major themes Were major themes clearly presented in the findings? | Yes |
